# *In vitro *cytotoxicity of Manville Code 100 glass fibers: Effect of fiber length on human alveolar macrophages

**DOI:** 10.1186/1743-8977-3-5

**Published:** 2006-03-28

**Authors:** Patti C Zeidler-Erdely, William J Calhoun, Bill T Ameredes, Melissa P Clark, Gregory J Deye, Paul Baron, William Jones, Terri Blake, Vincent Castranova

**Affiliations:** 1 Health Effects Laboratory Division, National Institute for Occupational Safety and Health, Morgantown, WV, USA; 2 AAARC, Division of Pulmonary, Allergy, and Critical Care Medicine, University of Pittsburgh, Pittsburgh, PA, USA; 3 Division of Applied Research and Technology, National Institute for Occupational Safety and Health, Cincinnati, OH, USA; 4 Division of Respiratory Disease Studies, National Institute for Occupational Safety and Health, Morgantown, WV, USA

## Abstract

**Background:**

Synthetic vitreous fibers (SVFs) are inorganic noncrystalline materials widely used in residential and industrial settings for insulation, filtration, and reinforcement purposes. SVFs conventionally include three major categories: fibrous glass, rock/slag/stone (mineral) wool, and ceramic fibers. Previous *in vitro *studies from our laboratory demonstrated length-dependent cytotoxic effects of glass fibers on rat alveolar macrophages which were possibly associated with incomplete phagocytosis of fibers ≥ 17 μm in length. The purpose of this study was to examine the influence of fiber length on primary human alveolar macrophages, which are larger in diameter than rat macrophages, using length-classified Manville Code 100 glass fibers (8, 10, 16, and 20 μm). It was hypothesized that complete engulfment of fibers by human alveolar macrophages could decrease fiber cytotoxicity; i.e. shorter fibers that can be completely engulfed might not be as cytotoxic as longer fibers. Human alveolar macrophages, obtained by segmental bronchoalveolar lavage of healthy, non-smoking volunteers, were treated with three different concentrations (determined by fiber number) of the sized fibers *in vitro*. Cytotoxicity was assessed by monitoring cytosolic lactate dehydrogenase release and loss of function as indicated by a decrease in zymosan-stimulated chemiluminescence.

**Results:**

Microscopic analysis indicated that human alveolar macrophages completely engulfed glass fibers of the 20 μm length. All fiber length fractions tested exhibited equal cytotoxicity on a per fiber basis, i.e. increasing lactate dehydrogenase and decreasing chemiluminescence in the same concentration-dependent fashion.

**Conclusion:**

The data suggest that due to the larger diameter of human alveolar macrophages, compared to rat alveolar macrophages, complete phagocytosis of longer fibers can occur with the human cells. Neither incomplete phagocytosis nor length-dependent toxicity was observed in fiber-exposed human macrophage cultures. In contrast, rat macrophages exhibited both incomplete phagocytosis of long fibers and length-dependent toxicity. The results of the human and rat cell studies suggest that incomplete engulfment may enhance cytotoxicity of fiber glass. However, the possibility should not be ruled out that differences between human versus rat macrophages other than cell diameter could account for differences in fiber effects.

## Background

Synthetic vitreous fibers (SVFs) are inorganic noncrystalline materials widely used in residential and industrial settings for insulation, filtration, and reinforcement purposes. SVFs conventionally include three major categories: fibrous glass, rock/slag/stone (mineral) wool, and ceramic fibers [1]. The chemical composition of fibrous materials is known to play a role in fiber-induced toxicity as fiber biodurability directly correlates with pathogenic potential in rodents [2], but it has also been suggested that fiber length is an important factor. In the past, the study of fiber length as a cause of toxicity has been complicated by the inability to obtain pure size-selected fiber samples. However, the development of the dielectrophoretic classifier by Baron and colleagues has aided in the study of monodisperse size-selected fiber samples on lung cell activation and toxicity [3]. This classifier separates fibers by length using dielectrophoresis that involves the movement of neutral particles in a gradient electric field [3,4]. Rodent macrophage toxicity and activation have previously been demonstrated *in vitro *in our laboratory using these length-classified fibers and indeed, fiber length was an important determinant [5-7].

Frustrated or incomplete phagocytosis has been implicated as a mechanism of fiber-induced cytotoxicity. This process involves repeated attempts by a phagocyte to engulf a fiber longer than its diameter, thereby possibly enhancing its respiratory burst activity [8]. In comparison to short fibers that are fully engulfed, longer fibers may cause sustained cellular activation and increase phagocyte recruitment into the airspace, subsequently increasing lung oxidant burden [9-11]. Indeed, several *in vivo *and *in vitro *rodent studies suggest longer fibers are more pathogenic than short fibers [12-14]. However, macrophage size is relevant when investigating fiber toxicity because human alveolar macrophages are larger in size than rat alveolar macrophages, approximately 18 and 13 μm in average diameter, respectively [15]. Therefore, the purpose of this study was to examine the influence of fiber length on isolated primary human alveolar macrophages, which are larger in diameter than rat macrophages, using length-classified Manville Code 100 (JM-100) glass fibers (8, 10, 16, and 20 μm). Respiratory burst activity and leakage of cytosolic lactate dehydrogenase (LDH) were used as parameters of activation and toxicity, respectively. Microscopic analysis was also conducted to determine if frustrated phagocytosis had occurred. A comparison to results obtained using the rat alveolar macrophage is made. Since this investigation employed a static rather than a flow system, issues of fiber solubility were not addressed.

## Results

### Glass fiber induced LDH

**Figure 1:**
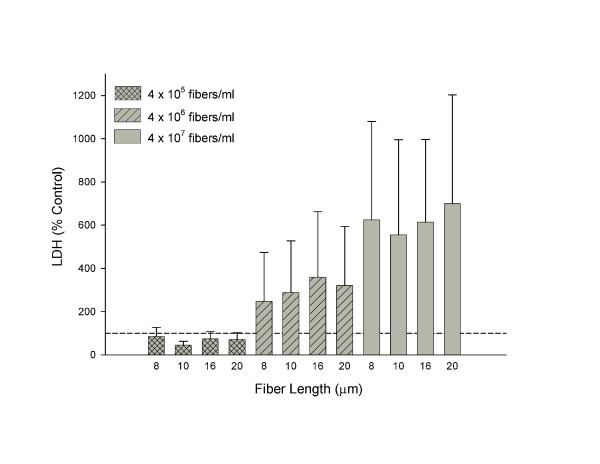
Lactate dehydrogenase release from primary human alveolar macrophages (1 × 10^5 ^cells/well) following exposure to JM-100 glass fibers for 18 hours. Data are presented as percent control from 100%. Bars represent mean values ± S.E. of three independent experiments. No significant difference was found among fiber length groups of a given concentration.

Figure 1 shows glass fiber-induced cytotoxicity on human alveolar macrophages as measured by the LDH assay 18 hours post-treatment *in vitro*. The fiber lengths tested (8, 10, 16, and 20 μm) all exhibited equal cytotoxic effects. These effects were not significantly different among fiber length groups of the same concentration, i.e., fiber length did not affect cytotoxicity over the range of lengths evaluated. A trend toward a concentration-response was observed although this did not achieve statistical significance. Table 1 depicts the effects of glass fiber length on rat alveolar macrophages as reported previously [7]. Significant toxicity, measured as % of total LDH, occurred with the long (17 μm) glass fiber, but a shorter fiber (7μm) showed little or no toxic effect.

**Table 1:** Relative Toxicity (% of total LDH) of Rat Alveolar Macrophages Exposed to Length Classified Glass Fibers

Fiber Length (μm)	Log (fiber #/ml)	
	
	10^6 ^(1:2.5 fiber:cell)	10^7 ^(4:1 fiber:cell)
3	ND	ND
4	ND	ND
7	ND	2%
17	19%*	45%*

*Note*: Adapted from Blake et al., 1998; ND is not detected ; LDH is lactate dehydrogenase; * represents a significant difference from control

### Glass fiber induced inhibition of zymosan-stimulated chemiluminescence

**Figure 2:**
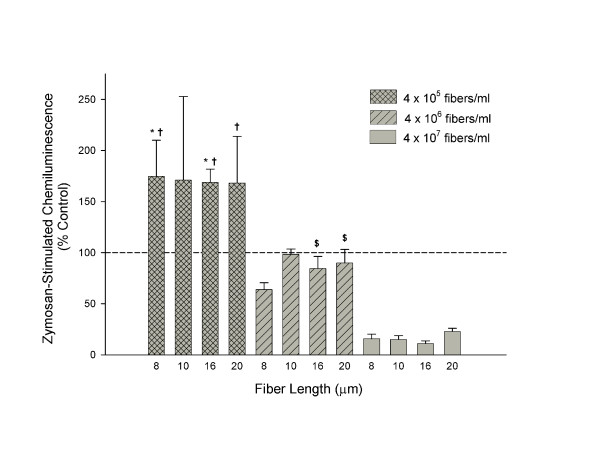
Inhibition of zymosan-stimulated primary human alveolar macrophage chemiluminescence following an18 hour exposure to JM-100 glass fibers. Data are presented as percent control from 100%. Bars represent mean values ± S.E of three independent experiments. * - indicates a significant difference between the lowest (4 × 10^5 ^fibers/ml or 1:1 fiber:cell) and intermediate (4 × 10^6 ^fibers/ml or 8:1 fiber:cell) fiber concentrations, †- between the lowest and highest (4 × 10^7 ^fibers/ml or 80:1 fiber:cell) fiber concentrations, and $ - between the intermediate and highest fiber concentrations of the same fiber length (p ≤ 0.05). Fiber length did not affect human alveolar macrophage function.

Zymosan-stimulated chemiluminescence, reported as % control, is depicted in Figure 2. Zymosan, a particulate β-glucan from yeast cell walls, was used to stimulate reactive species production in the human alveolar macrophages exposed to glass fibers for 18 hours *in vitro*. Data show a concentration-dependent decrease in macrophage activation (i.e. reactive species production) with increasing fiber concentration. Fiber length did not significantly affect human alveolar macrophage function. It is of interest to note the lowest concentration of fibers caused a priming (71% increase in activation) of the human cells to release reactive species in response to zymosan while the highest concentration caused an 84% loss of activation. Table 2, adapted from Blake et al., represents the percent decreased activation versus control observed in rat macrophages following glass fiber exposure for 18 hours [7]. In contrast to the human cells, the rat macrophages show distinctly decreased oxidative function with increasing fiber length, with the 17 μm fiber exhibiting a stronger effect (100% loss of activation) than the 7μm fiber (15% reduction of activation). In addition, no cellular priming was observed in the rat macrophages as compared to the human.

**Table 2:** Relative Toxicity (% Decreased Activation vs. Control) of Rat Alveolar Macrophages Exposed to Length Classified Glass Fibers

Fiber Length (μm)	Log (fiber #/ml)	
	
	10^6 ^(1:2.5 fiber:cell)	10^7 ^(4:1 fiber:cell)
3	ND	ND
4	ND	ND
7	ND	15%
17	53%*	100%*

*Note*: Adapted from Blake et al., 1998; ND is not detected;* represents a significant decrease from control

### Microscopic examination of primary human alveolar macrophages

**Figure 3:**
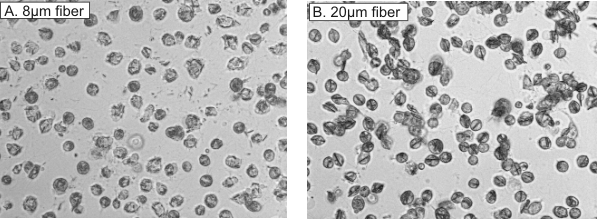
Microscopic analysis of primary human alveolar macrophages engulfing glass fibers. Panel A demonstrates the ability of the alveolar macrophages to engulf 8 μm glass fibers. Panel B verifies effective phagocytosis of 20 μm glass fibers by human alveolar macrophages.

Figure 3, panel A, demonstrates the ability of human macrophages to engulf 8 μm glass fibers *in vitro*. Figure 3, panel B, demonstrates that human macrophages can successfully engulf 20 μm glass fibers. Data from Blake et al. revealed incomplete phagocytosis by rat alveolar macrophages occurred with glass fibers ≥ 17 μm in length [7]. These results suggest human alveolar macrophages can completely phagocytize fiber lengths that rat alveolar macrophages can not. Therefore, frustrated phagocytosis and its possible effects do not appear to be a factor with human alveolar macrophages exposed to fibers ≤ 20 μm in length.

## Discussion

The present study employed an *in vitro *system to expose primary human alveolar macrophages to monodisperse JM-100 glass fibers of different target lengths. Using this system, human alveolar macrophages were assessed for length-dependent cellular effects and results were compared to previous data obtained with rat alveolar macrophages. The data presented here reveal differences in the responses of rat versus human macrophages to glass fibers of various lengths.

Previous studies have shown that rat alveolar and mouse peritoneal macrophages attempt to engulf glass fibers and that successful phagocytosis is influenced by fiber length [5,7]. Short (7 μm) glass fibers were completely engulfed, whereas long (17 μm) glass fibers were only partially engulfed. Human alveolar macrophages have a larger diameter than the rat counterpart; therefore, it was hypothesized that these phagocytes would completely engulf longer fibers and the absence of frustrated phagocytosis would attenuate cytotoxicity. Overall, the data supported this hypothesis.

Human alveolar macrophage function, measured as zymosan-stimulated chemiluminescence, was significantly affected by fiber concentration but not fiber length over the range of 8–20 μm (Figure 2). The lowest of three fiber concentrations (4 × 10^5 ^fibers/ml or 1:1 fiber:cell ratio) primed the cellular response to zymosan while the highest concentration (4 × 10^7^fibers/ml or 80:1 fiber:cell ratio) decreased this response from control. The activation level of cells exposed to the intermediate fiber concentration (4 × 10^6 ^fibers/ml or 8:1 fiber:cell ratio) was similar to that of controls). In contrast, our previous findings showed no increased priming effect of JM-100 glass fibers in rat alveolar macrophages at any concentration. This differential response between human and rat cells may be the result of several possible factors. First, fiber to cell ratio has been shown in our laboratory to be important in the activation of rodent macrophages. Prior studies revealed a fiber to cell ratio of at least 5:1 is required for significant tumor necrosis factor-α (TNF-α) production in mouse macrophages [5]. The effective fiber to cell ratio found to elicit human macrophage cellular priming in this study was 1:1. Approximately a 1:2.5 fiber to cell ratio (10^6 ^fibers/ml) was used as a low concentration in the rat macrophage studies reported by Blake et al., which may not have been high enough to prime the cellular response to zymosan above control following short fiber phagocytosis [7]. Secondly, luminol was used as the light enhancer in the present study, but lucigenin was used in the rat cell study. Luminol detects multiple reactive species compared to lucigenin, which is a superoxide specific light enhancer [16-18]. Most likely, superoxide is not the sole oxidant produced in glass fiber-exposed cells. Therefore, this experimental system may have an enhanced detection level compared to the past system. Finally, the result may reflect inherent differences in the oxidant production of rat and human alveolar macrophages. Human alveolar macrophages are reportedly more active than the rat in the production of reactive species following particulate exposure [19]. Whether this is the case with glass fibers would need further investigation.

It was found that no fiber length or concentration induced significant cellular membrane damage as assessed by cytosolic LDH release into the cellular supernatant although a concentration-dependent trend was apparent (Figure 1). Castranova et al. reported effects of metallic ions on particle-stimulated oxygen consumption and chemiluminescence in alveolar macrophages, i.e., functional assays, occurs at concentrations that do not affect the integrity of the cellular membrane [20]. Therefore, a reason for the lack of a significant LDH concentration-response may be that the cellular function is typically affected before membrane damage making the chemiluminescence response a more sensitive measure of cytotoxic effects.

In summary, this study reported no effects of fiber length over the range of 8–20 μm on the human alveolar macrophage response. This conflicts with previous data where increased glass fiber concentration decreased chemiluminescence and increased LDH in rat alveolar macrophages but major length-dependent effects were evident. The reason for this discrepancy most likely is due to the larger diameter of human alveolar macrophages, resulting in the lack of frustrated phagocytosis in the present experimental system. Microscopic analysis verified these cells were able to completely engulf even the longest fiber sample tested (20 μm) (Figure 3B). Blake et al. demonstrated cellular effects on rat alveolar macrophages (diameter ~13 μm) using 17 and 33 μm glass fibers, approximately 4–20 μm longer than the cells (multiple rat macrophages were often observed adhered to a long fiber with the fiber appearing to protrude through the cellular membrane) [7]. In the present study, the longest fiber sample used was 20 μm, only 2 μm longer than the human alveolar macrophage cellular diameter; therefore not a sufficient length to cause frustrated phagocytosis. Consequently, further studies are needed using fiber samples over a range of 20–40 μm in length. Assuming similar mechanisms are involved in human and rat fiber phagocytosis, results most likely would agree with those reported by Blake and colleagues.

## Conclusion

This study showed: 1.) an absence of length-associated cytotoxicity in primary human alveolar macrophages that was previously observed in rat alveolar macrophages treated *in vitro *with length-classified glass fibers 2.) a possible mechanism for this absence of length-dependent cytotoxicity may be the lack of frustrated phagocytosis in the human macrophages versus the rat 3.) human alveolar macrophages appear to be activated by glass fibers of monodisperse lengths at a fiber to cell ratio of approximately 1:1, while significant cytotoxicity was observed only at an excessively high fiber:cell ratio of 80:1. In conclusion, the use of monodisperse length-classified fiber samples will aid in the determination of specific fiber lengths that macrophages can engulf. In addition, these preliminary data may aid in the design of future *in vivo *experiments using fibrous particles.

## Methods

### Fiber samples

Bulk samples of JM-100 glass (Manville code 100 supplied by John Mansville Corporation) were first milled, aerosolized, and separated into length categories using dielectrophoresis as previously described [3,4]. The dielectrophoretic classifier was operated in a differential mode so that fibers with narrow length distributions were extracted in an air suspension at the end of the classifier. These length-classified fiber samples were collected on polycarbonate (Nuclepore) filters at rates up to 1 mg/day. Fibers were scraped off the filters for microscopic analysis and for biological experiments.

Samples of the length-classified fibers were prepared for size and count analysis by adding weighed portions of the dusts to freshly filtered water. These samples were then diluted and filtered through polycarbonate filters. Measurements of length, width, and fiber count/mass were made using a JEOL JSM-6400 scanning electron microscope (Table 3). Measurements at each magnification were referenced to a National Institute of Standards and Technology electron microscopy standard rule.

**Table 3:** Physical Characteristics of JM-100 Glass Fibers Determined by SEM

Measurement	Length Classified Sample			
	
	8 μm	10 μm	16 μm	20 μm
Length^a ^(μm)	8.38 ± 2.89	10.40 ± 4.63	16.22 ± 1.90	18.90 ± 2.64
Width (μm)	0.69 ± 0.29	0.70 ± 0.29	1.08 ± 0.43	0.97 ± 0.47
Fiber #/mg	11.4 × 10^7^	8.95 × 10^7^	2.2 × 10^7^	2.92 × 10^7^

^a^Length measurements reported for the 16 and 20 μm fiber samples exclude short fiber populations that occasionally adhered to the long fibers during classification. Prior to use, fiber suspensions were gently vortexed rather than sonicated to minimize release of these short fibers. Inclusion of short fibers in the analysis slightly lowered the mean fiber length in the distribution for the 16 and 20 μm fiber samples. *Note*: Values for length and width are means ± SD

All fiber samples were heat-sterilized and stored at 4–6°C. Prior to each experiment, the fibers were suspended in sterile Ca^+2 ^+ Mg^+2 ^free phosphate-buffered saline (PBS).

### Human bronchoalveolar lavage

Human alveolar macrophages were collected by a standard procedure at University of Pittsburgh. Briefly, a total of six male healthy subjects between the ages of 20 and 40 years were recruited for study. All signed a statement of informed consent approved by the University of Pittsburgh Institutional Review Board for Biomedical Research. Subjects were screened by history, physical examination, and spirometry. All subjects had to exhibit normal spirometry, and have no history of asthma or allergic diseases. Skin test responses to a panel of 15 common allergens, plus histamine phosphate as a positive control (Greer Laboratories, Lenoir, NC), were determined using the prick-puncture method. Any subject demonstrating a positive skin test to an allergen was deemed to have atopic disease and was excluded. Bronchoscopy and bronchoalveolar lavage (BAL) were performed according to published methods [21-23]. Briefly, subjects were lightly sedated with midazolam 1.0 mg and atropine 0.5 mg IM, and meticulous local anesthesia of the nasopharynx was obtained using 4% cocaine solution, 1% lidocaine by gargle, and 1% benzocaine aerosol. Additional topical 1% lidocaine, via the bronchoscope working channel, was applied to the vocal cords and airway mucosa. Total lidocaine dose delivered below the vocal cords was in all cases less than 200 mg. The bronchoscope was advanced to the right upper lobe, and wedged in a subsegment of the anterior segment. BAL was then performed using two aliquots of sterile saline solution (0.9% NaCl, 37°C, 60 ml each), recovered by hand suction. The bronchoscope was then moved to a subsegment of the right middle lobe, medial segment, and BAL was again performed. Cells were recovered from BAL fluid by centrifugation at 400 × g for 15 minutes and the resulting cell pellet was washed once with Hanks Balanced Salt Solution (Sigma, St Louis). Cells were counted using a hemocytometer and adjusted to 2 × 10^6 ^cells/ml in Hanks Balanced Salt Solution. Differential cell counts were performed on Diff-Quick stained cytocentrifuge slides, and ≥300 cells were identified as alveolar macrophages, lymphocytes, eosinophils, or neutrophils. Viability by trypan blue exclusion always exceeded 95%, and cell populations were in excess of 95% alveolar macrophages, with the balance of the population comprised of lymphocytes. Neutrophils were rarely observed as were eosinophils. In healthy volunteers, purification over discontinuous Percoll gradients is unnecessary [23].

### Primary human alveolar macrophage zymosan-stimulated chemiluminescence

Human alveolar macrophages were plated in a white opaque 96-well plate at 1 × 10^5 ^cells/well in 200 μl of sterile Eagle's modified essential medium (Biowhittaker, Walkersville, MD) without phenol red supplemented with 2% heat-inactivated fetal bovine serum, 1 mM glutamine, 100 units/ml penicillin/streptomycin, and 10 mM HEPES at pH 7.2. After a 2 hour incubation at 37°C, the adherent cells were washed three times with warm media. Glass fibers (8,10,16, or 20 μm) were then added to the cells at three different concentrations determined by fiber number: 4 × 10^5^, 4 × 10^6^, and 4 × 10^7 ^fibers/ml. Cultures were brought to a final volume of 200 μl and each treatment was done in duplicate. After 18 hours at 37°C, the plate was centrifuged and the supernatant harvested and kept on ice for later analysis. Warm HEPES-buffered solution (pH = 7.4) was then added to the alveolar macrophages on the plate and zymosan-stimulated chemiluminescence was measured. The lumigenic substance luminol (1 mM) was added to all wells of the assay plate followed by zymosan (2 mg/ml) or HEPES-buffered solution to a final volume of 220 μl/well. Chemiluminescence was measured at 460 nm using a microplate chemiluminometer (ML 3000 Microtiter Plate Luminometer, Dynatech Laboratories, Chantilly, VA) for 30 minutes and total cpm per well per 30 minutes was recorded. Zymosan-stimulated chemiluminescence was calculated as the stimulated (with zymosan) value minus the corresponding unstimulated (without zymosan) value.

### Lactate dehydrogenase activity

LDH activity was determined in the culture supernatant by monitoring the LDH catalyzed oxidation of pyruvate coupled with the reduction of NAD at 340 nm using a commercial kit and a Cobas Mira Plus Transfer Analyzer (Roche Diagnostics Systems, Montclair, NJ).

### Microscopic analysis

Photographs of human alveolar macrophages were taken using an Olympus IX70 inverted light microscope equipped with a Dage camera.

### Data analysis

All data were analyzed using a one-way analysis of variance model (ANOVA) with a significant difference defined as p ≤ 0.05. Pairwise differences were assessed through appropriate contrasts.

## Abbreviations

BAL Bronchoalveolar Lavage

JM-100 Manville Code 100 Glass Fibers

LDH Lactate Dehydrogenase

ND Not Detected

PBS Phosphate Buffered Saline

SEM Scanning Electron Microscope

SVFs Synthetic Vitreous Fibers

## Competing interests

The author(s) declare that they have no competing interests.

## Authors' contributions

PCZE carried out the *in vitro *cytotoxicity studies, analyzed the data, and drafted the manuscript. WJC, BTA, and MPC provided the human macrophages and provided assistance in study design and coordination. GJD and PB provided the length-classified fiber samples and aided in fiber analysis. WJ performed the fiber analysis and provided assistance in the study design. TB conducted studies with the rat alveolar macrophages. VC conceived of the study, participated in its design and coordination and helped in manuscript preparation. All authors read and approved the final manuscript.

## Disclaimer

The findings and conclusions in this report are those of the authors and do not necessarily represent the views of the National Institute for Occupational Safety and Health.
